# Establishing consensus on nutrition competencies for medicine: a Delphi study

**DOI:** 10.1136/bmjnph-2023-000807

**Published:** 2024-02-05

**Authors:** Breanna Lepre, Kylie J Mansfield, Sumantra Ray, Eleanor J Beck

**Affiliations:** 1 School of Medical, Indigenous and Health Sciences, University of Wollongong, Wollongong, New South Wales, Australia; 2 School of Human Movement and Nutrition Sciences, The University of Queensland, St Lucia, Queensland, Australia; 3 NNEdPro Global Institute for Food, Nutrition and Health St John’s Innovation Centre, NNEdPro, Cambridge, UK; 4 Graduate School of Medicine, University of Wollongong, Wollongong, New South Wales, Australia; 5 Fitzwilliam College, University of Cambridge, Cambridge, UK; 6 School of Biomedical Sciences, Ulster University, Coleraine, UK; 7 School of Health Sciences, University of New South Wales, Sydney, New South Wales, Australia

**Keywords:** Nutritional treatment, Preventive counselling

## Abstract

**Background:**

Significant research, regulatory bodies and even governmental resolutions have identified meaningful nutrition education for medical and other healthcare professionals as a priority. Doctors are well placed to provide nutrition care, yet nutrition education in medicine remains inadequate regardless of country, setting, or year of training. There remains a need to establish an accepted benchmark on nutrition competencies for medicine, as without consensus standards there is little likelihood of uniform adoption.

**Objective:**

This study aimed to establish consensus on nutrition competencies using a Delphi process to inform a framework for nutrition education in medicine.

**Methods:**

A three-round modified online Delphi survey of experts in healthcare practice, education and training, and experts by experience (service users) was conducted to provide a comprehensive consensus on nutrition competencies for medical practice.

**Results:**

Fifty-two experts (15.1% response rate) participated in Round 1, 42 completed Round 2 and 47 completed Round 3. Participants included medical professionals, dietitians, academics working in health professions education and policymakers from Australia, New Zealand, the UK and Northern Ireland. Twenty-seven service users (57.5% response rate) completed the Round 1 questionnaire, 19 completed Round 2 and 16 completed Round 3. By consensus, 25 nutrition competencies for medicine were defined. The service user panel identified an additional seven skills and attributes considered important in the receipt of nutrition care. Competencies that achieved consensus broadly fell into themes of team-based care, communication, professionalism (eg, attributes) and health promotion and disease prevention. This informs broad skills that may be taught in a nutrition context but could be included in other domains.

**Conclusions:**

The findings suggest doctors need the knowledge and skills to consider the findings from nutrition screening and assessment, coordinate nutrition care when an individual may benefit from further assessment or intervention and provide support for advice delivered by other experts as part of a multidisciplinary approach.

**WHAT IS ALREADY KNOWN ON THIS TOPIC**

- Nutrition education in medical training remains inadequate at all levels internationally. However, without consensus standards, there is little likelihood of uniform adoption.

**WHAT THIS STUDY ADDS**

- This is the first study to provide a consensus on nutrition competencies for medical practice in Australia, New Zealand, the UK and Northern Ireland.

**HOW THIS STUDY MIGHT AFFECT RESEARCH, PRACTICE OR POLICY**

- A total of 25 nutrition competencies were validated by expert consensus, which may provide guidance to educators and regulators of medical education. Nutrition education should be vertically integrated throughout the medical training process, provide opportunities for interprofessional development and be embedded within sociocultural frameworks to support the delivery of person-centred nutrition care.

## Introduction

Overweight, obesity and diet-related non-communicable diseases carry significant personal and financial burden and are a threat to achieving the United Nations’ Sustainable Development Goals. National policy, such as the Australian National Preventive Health Strategy, underscores the need for a healthcare workforce competent in nutrition.^1^ Doctors are well placed to support nutrition care, in part due to regular contact with the individuals for whom they provide care. In Australia, around 85% of the population sees a general practitioner (GP) at least once each year, and international studies estimate that 16%–24% of GP consultations feature some aspect of nutrition.^2 3^ In the secondary care setting, rates of malnutrition remain high, averaging 35% internationally.^4^ Despite this, nutrition education in medicine remains inadequate regardless of country, setting, or year of training.^5^

The need to enhance nutrition education for medical professionals has appeared in the literature for more than five decades, with seemingly little progress.^5^ The McGovern Resolution on Nutrition Education in Medical Schools (2022) identified meaningful nutrition education for medical and other healthcare professionals as a priority.^6^ In order to incentivise inclusion of nutrition in medical education, it may need to be embedded within curriculum or accreditation requirements and be part of relevant regulatory frameworks. The use of a competency-based approach in enhancing medical nutrition education has been previously established and has been shown to improve the ability to integrate nutrition into patient care.^7^ Recent developments in this space, such as the Association for Nutrition United Kingdom Undergraduate Curriculum for Nutrition for Medical Doctors, published in 2021, and the European Society for Clinical Nutrition and Metabolism position paper on nutrition education in medical schools, aimed to identify minimum requirements for nutrition in undergraduate medical education.^8 9^ However, without consensus standards, there is little likelihood of uniform adoption. This study brings together our previous work on published curriculum guidance and regulatory frameworks,^10 11^ and qualitative investigation of a medical and patient perspective on nutrition knowledge, skills and attributes relevant to doctors.^12 13^ This data were used, together with best practice in competency framework development to inform a framework for nutrition education in medicine. The final step in the development of this framework was to seek consensus on the competency items. Therefore, this study aimed to establish consensus on nutrition competencies using a Delphi process.

## Methods

**Figure 1:**
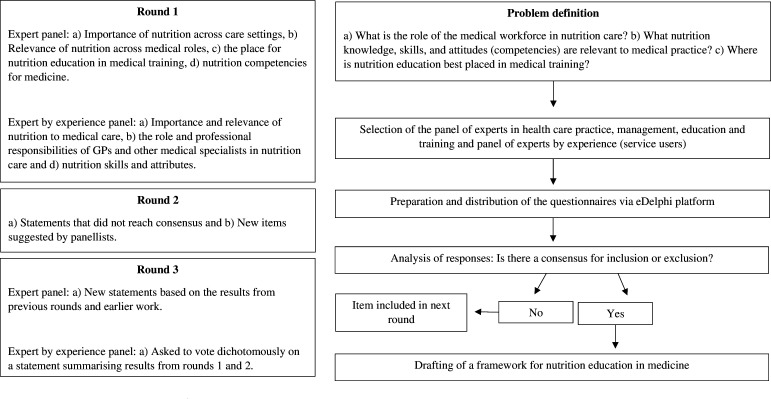
Schematic of the Delphi process. GPs, general practitioners.

We administered a three-round modified online Delphi (eDelphi) survey to systematically gather the opinions of a panel of experts in healthcare practice, management, education and training and experts by experience (service users) to identify consensus on nutrition competencies for medicine. For the purpose of this study, we defined a competency as a measure used to describe the idealised capacity of an individual to perform a role or set of tasks.^14^ Figure 1 shows the scheme used in this research.

The Delphi method consists of a series of iterative questionnaire rounds among a panel of experts, whereby consensus is built through a process of informed decision-making and has been used widely in the development of competency frameworks for health professions.^15^ The logic of the process is that combined numerical estimates of opinions would, in general, lead to more reliable estimates than those obtained from an individual. The Delphi method has been used widely in healthcare research, particularly where there is a lack of objective evidence to support decision-making on a clearly defined topic.^15^

### Recruitment

Seeking consensus from a range of perspectives has been shown to increase the validity of a competency framework.^12^ Thus, this study aimed to engage with a variety of experts in healthcare practice and service users (deemed ‘experts by experience’) to provide an all-round consensus on nutrition competencies for medicine, not just according to the medical profession themselves. Participants were recruited from Australia, New Zealand, the UK and Northern Ireland, considered together as they have similar models of medical education and healthcare systems. For example, healthcare systems in Australia, New Zealand and the UK are based on the ideology of an accessible and publicly funded healthcare system, and while the organisation of medical specialist care and access to nutrition services may vary across and between countries, primary care services are provided mainly by GPs. As there is little to no agreement as to what defines an ‘expert’, defined criteria were used to reduce the risk of selection bias (table 1).

**Table 1:** Selection criteria for Delphi participants

Group	Selection criteria
Experts in healthcare practice (including dietitians, nurses or doctors)	Bachelor’s degree or higher in nutrition, medicine or nursing Professional practice experience in the field of nutrition, medicine or nursing High motivation and willingness to participate in the study.
Experts in healthcare education and training	Considered a leader in medical or nutrition education and/or training (eg, responsible for medical training) Professional experience in education and/or training in medicine or nutrition High motivation and willingness to participate in the study.
Experts by experience (service users)	End user of healthcare services High motivation and willingness to participate in the study.

An email contact list for experts was developed, primarily through existing professional networks, including the NNEdPro Global Institute for Food, Nutrition and Health, networks of the research team, snowball sampling and a list of contacts developed by internet search of websites of universities in Australia and the UK, and public health and Government websites. A targeted email was also sent to dietitians who were credentialed as ‘advanced’ or as ‘fellows’ in Australia. Information about the study was posted to The Royal Australian College of General Practitioners (RACGP) research noticeboard. An email contact list was also prepared for the panel of experts by experience (hereafter ‘service users’), primarily through existing networks of the research team and social media. Participants who accepted the invitation (ie, self-selected participation) were directed to the first questionnaire via a link in the email invitation. A reminder email with an invitation to participate in round 1 was sent after 1 week in an attempt to maximise participation. Participants who accepted the invitation for round 1 were invited to participate in consecutive rounds.

### Survey instruments

Preliminary statements were informed by a review of published nutrition competencies for medicine, interviews with doctors and focus groups with service users.^10^ Three rounds (February–May 2022) of questionnaires were developed utilising a web-based survey tool (eDelphi 2022, www.eDelphi.org) and completed via this platform. Participants had an initial 2 weeks to complete each round of the survey and reminders were sent by email at 1 week before the end of the response period. At the end of each round, a result report was prepared and made available to participants. Feedback and discussion were conducted among researchers at the end of each round. All questionnaires included an option to provide open-text comments, shown to generate valuable data and increase the credibility of subsequent frameworks.^16^ The questionnaires from each round are included in online supplemental material.

10.1136/bmjnph-2023-000807.supp1 Supplementary data

For the expert panel, statements were related to (1) the importance of nutrition across care settings (eg, primary medical care), (2) the relevance of nutrition care across medical roles (eg, resident or specialist), (3) professional responsibilities in nutrition care and (4) where nutrition education might best fit into medical training.

A separate three-round modified Delphi survey was sent to service users, including (1) the relevance and importance of nutrition to patient medical care and (2) the skills and attributes that may be important for provision of nutrition care.

The questionnaires for round 2 included competencies that did not achieve consensus in round 1, with slight changes to wording where suggested as well as additional competencies suggested by panellists.

### Data analysis

Responses to each round of Delphi surveys were exported to Microsoft Excel Spreadsheet Software for storage and analysis by the primary author. Data were analysed using basic descriptive statistics and expressed as percent agreement, percent disagreement, mean, IQR and SD. A predefined level of agreement (for inclusion or exclusion) was used in the absence of a uniform definition for consensus.^17^ The consensus for inclusion was defined as ≥80% agreement (Likert scale 4 and 5), and for exclusion, ≥50% disagreement (Likert scale 1 and 2) or ≤50% agreement among panellists. Items that achieved consensus were not considered further and were excluded from following survey rounds.

## Results

### Expert panel

A total of 344 individuals based in Australia, New Zealand, the UK and Northern Ireland were formally invited to participate in the expert panel. Fifty-two experts (15.1% response rate) participated in round 1, 42 completed round 2 and 47 completed round 3. The expert sample was predominantly men, aged between 26 and 72 years and included medical professionals, dietitians, academics working in health professions education and policymakers (table 2).

**Table 2:** Demographics of Delphi expert panellists

Expert panel			
Characteristics	Completed round 1 (n=52)	Completed round 2 (n=42)	Completed round 3 (n=47)
Age (years)			
Mean	49.9	52.7	51.9
Range	46 (26–72)	47 (26–73)	46 (27–73)
Gender			
Female	21 (41.2%)	16 (39.0%)	18 (37.5%)
Male	30 (58.8%)	25 (61.0%)	30 (62.5%)
Non-binary/prefer not to say	0	0	0
Country			
Australia	22 (59.5%)	22 (66.7%)	24 (63.2%)
New Zealand	12 (32.4%)	6 (18.2%)	10 (26.3%)
United Kingdom and Northern Ireland	2 (5.4%)	4 (12.1%)	4 (10.5%)
Other	1 (2.7%)	1 (3.0%)	0
Role			
Medical practitioner	18 (35.3%)	14 (34.2%)	17 (35.4%)
Dietitian	21 (41.2%)	17 (41.5%)	18 (37.5%)
Academic working in health professions education	10 (19.6%)	10 (24.4%)	12 (25.0%)
Other (eg, policy maker)	2 (3.9%)	0	1 (2.1%)
Other health professional (eg, nurse)	0	0	0
Manager or employer in healthcare	0	0	0
Medical specialty			
General practice	9 (50.0%)	8 (57.1%)	8 (47.1%)
Public health medicine	1 (5.6%)	1 (7.1%)	1 (5.9%)
Intensive care	1 (5.6%)	1 (7.1%)	1 (5.9%)
Geriatrics	1 (5.6%)	1 (7.1%)	2 (11.8%)
General surgery	1 (5.6%)	0 (0.0%)	1 (5.9%)
Hepatology	1 (5.6%)	0 (0.0%)	0 (0.0%)
Ophthalmology	1 (5.6%)	0 (0.0%)	0 (0.0%)
Palliative care	1 (5.6%)	0 (0.0%)	1 (5.9%)
Obstetrics and gynaecology	1 (5.6%)	1 (7.1%)	1 (5.9%)
Endocrinology	1 (5.6%)	0 (0.0%)	1 (5.9%)
Did not provide specialty	0 (0.0%)	2 (14.3%)	1 (5.9%)
Postgraduate nutrition education			
Yes	25 (49.0%)	25 (61.0%)	26 (54.2%)
Diploma, certificate or other	8	6	6
Bachelors or masters	7	10	9
PhD	12	10	12
No	26 (51.0%)	16 (39.0%)	22 (45.8%)

### Nutrition in medical education and care

There was agreement in round 1 that nutrition is important across all care settings, though primary care was identified as the ideal setting in the context of disease prevention. The perceived relevance of nutrition care increased across the continuum of roles in medical practice, ranging from 76.5% for medical students to 94.1% for general practice registrars and specialist GPs. The majority (65.9%) of panellists indicated that the primary role of a doctor in nutrition care is to coordinate care, though there was some agreement that their role may also include nutrition assessment and brief dietary advice. Few panellists indicated that doctors do not have a role in nutrition care, or that they should be the main provider of said care (table 3 and box 1). Taken together, these results may indicate that the role of a doctor in nutrition care includes nutrition screening and/or assessment, and the coordination of nutrition care for those who may benefit from further assessment or specialist advice. Panellists agreed that nutrition education is best placed in medical school (77.1% level of agreement). There was otherwise limited consensus on where nutrition is best placed in medical training, though the findings indicate some agreement that nutrition education may be less relevant other registrar/specialty training and in ongoing continuing professional development (table 4 and box 2).

**Table 3:** Level of agreement for the role of a doctor in nutrition care

Statement	Mean	Rank order (n, %)*					
		1	2	3	4	5	6
The primary role of a doctor in nutrition care is to **coordinate**, which might include nutrition screening and assessment, identifying a nutrition-related issue and arranging a referral to a dietitian or other service as well as reinforcing recommendations from other professionals (eg, a dietitian or diabetes educator).	1.532	29, 65.9%	10, 22.7%	4, 9.1%	1, 2.3%	0, 0.0%	0, 0.0%
The primary role of a doctor in nutrition care is to **assess** nutrition status, which might include nutrition screening and assessment, identifying a nutrition-related issue and providing brief dietary advice.	2.617	2, 4.5%	22, 50.0%	11, 25.0%	8, 18.2%	1, 2.3%	0, 0.0%
The primary role of a doctor in nutrition care is to be **person-centred**, which might include the provision of nutrition care if the patient requests it or if it is relevant to the medical care being provided.	2.702	10, 22.7%	10, 22.7%	11, 25.0%	9, 20.5%	4, 9.1%	0, 0.0%
The primary role of a doctor in nutrition care is to **educate**, which might include nutrition screening and assessment, identifying a nutrition-related issue and the provision of nutrition education to patients.	3.511	2, 4.5%	1, 2.3%	15, 34.1%	22, 50.0%	4, 9.1%	0, 0.0%
A doctor should be the **main provider of nutrition care**, which might include nutrition screening and assessment, identifying a nutrition-related issue, the provision of a nutrition care plan, nutrition education and arranging a follow-up appointment to monitor progress.	5.128	0, 0.0%	1, 3.3%	0, 0.0%	3, 6.8%	28, 63.6%	12, 27.3%
Nutrition care is **not part of the role of a doctor**. Dietitians/nutritionists are the specialists in nutrition.	5.511	1, 3.3%	0, 0.0%	3, 6.8%	1, 3.3%	7, 15.9%	32, 72.7%

*1 = resonates most, 6 = resonates least.

Box 1 **Rank-order of statements related to the role of a doctor in nutrition care**

- The primary role of a doctor in nutrition care is to coordinate, which might include nutrition screening and assessment, identifying a nutrition-related issue and arranging a referral to a dietitian or other service as well as reinforcing recommendations from other professionals (eg, a dietitian or diabetes educator).
- The primary role of a doctor in nutrition care is to assess nutrition status, which might include nutrition screening and assessment, identifying a nutrition-related issue and providing brief dietary advice.
- The primary role of a doctor in nutrition care is to be person centred, which might include the provision of nutrition care if the patient requests it or if it is relevant to the medical care being provided.
- The primary role of a doctor in nutrition care is to educate, which might include nutrition screening and assessment, identifying a nutrition-related issue and the provision of nutrition education to patients.
- A doctor should be the main provider of nutrition care, which might include nutrition screening and assessment, identifying a nutrition-related issue, the provision of a nutrition care plan, nutrition education and arranging a follow-up appointment to monitor progress.
- Nutrition care is not part of the role of a doctor. Dietitians/nutritionists are the specialists in nutrition.

**Table 4:** Level of agreement for where nutrition is best placed in medical education

Stage of medical education	Mean	Rank* (n,%)					
		1	2	3	4	5	6
Medical school	1.588	37, 77.1%	4, 8.3%	1, 2.1%	2, 4.2%	2, 4.2%	2, 4.2%
Foundation/intern training	3.392	3, 6.3%	17, 35.4%	7, 14.6%	5, 10.4%	8, 16.7%	8, 16.7%
Residency	3.941	2, 4.2%	4, 8.3%	14, 29.2%	9, 18.8%	12, 25.0%	7, 14.6%
Specialist GP training	3.529	1, 2.1%	11, 22.9%	12, 25.0%	15, 31.3%	8, 16.7%	1, 2.1%
Other registrar/specialist training	4.529	1, 2.1%	4, 8.3%	8, 16.7%	8, 16.7%	14, 29.2%	13, 27.1%
Continuing professional development (CPD)	4.020	4, 8.3%	8, 16.7%	6, 12.5%	9, 18.8%	4, 8.3%	17, 35.4%

*1 = best, 6 = least preferred.

GP, general practitioner.

Box 2 **Rank-order of where nutrition education is best-placed in medical training (1=best; 6=least preferred)**

- Medical school.
- Foundation/intern training.
- Residency.
- Specialist general practitioner training.
- Ongoing continuing professional development.
- Other registrar/specialty training.

### Nutrition competencies for medicine

**Figure 2:**
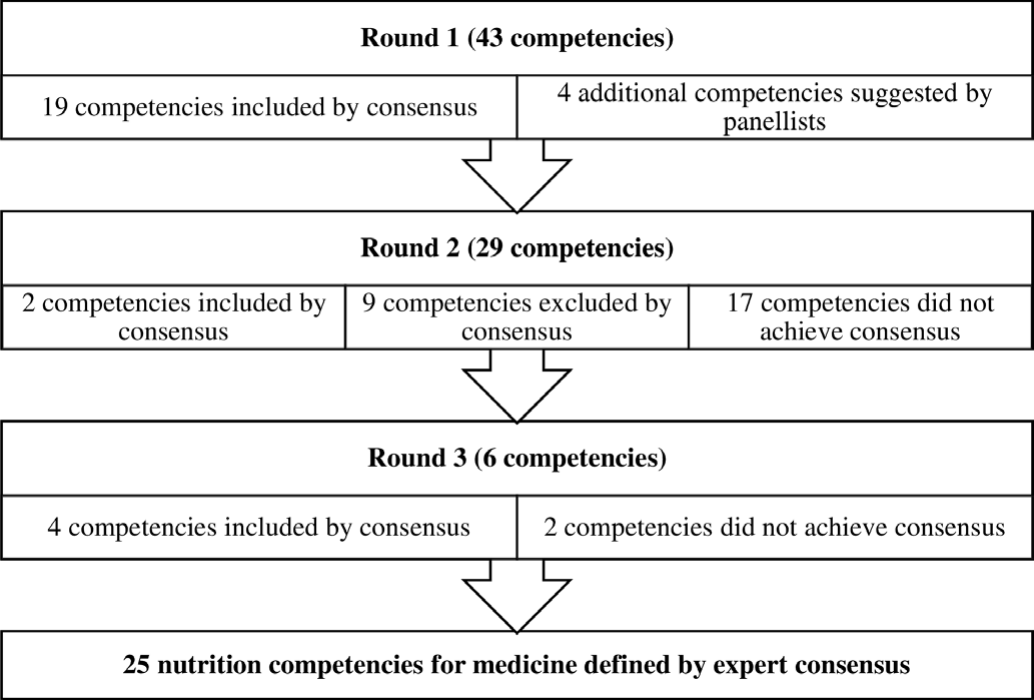
Consensus results across Delphi rounds.

A total of 25 nutrition competencies for medicine achieved consensus across the Delphi process (table 4), with the majority (19) achieving consensus in round 1 (figure 2). In round 1, panellists suggested additional items related to food and health systems and environmental sustainability, and the role of diet in mental health, among others (online supplemental materials).

Overall, competencies related to multidisciplinary care achieved a high level of agreement (box 3). For subsequent rounds, researchers considered where varied results were achieved and how these might be combined or split to better discriminate opinions between experts. For example, ‘nutrition screening’ had a relatively high level of agreement across rounds 1 and 2 (75.0% and 64.1%, respectively), while ‘use a validated tool to conduct nutrition screening and assessment’ had a lower level of agreement among panellists (66.7% and 51.3% in rounds 1 and 2). Only 36.4% of doctors agreed that medical professionals should be able to conduct nutrition screening and assessment, compared with 56.3% of dietitians and 60.0% of academics. In contrast, only 50.0% of dietitians agreed that a medical professional should be able to identify and define nutritional problems, compared with 81.8% of doctors and 100.0% of academics. Based on these results, panellists were asked to rate new items related to elements of nutrition screening and assessment in round 3 (online supplemental materials). Greater than 80% of panellists agreed that a medical professional might ‘consider the findings from nutrition screening and assessment as part of medical care’, ‘coordinate care when an individual may benefit from further nutrition assessment or specialist dietary advice’ and ‘reinforce nutrition advice or recommendations provided by a specialist (eg, a dietitian)’ (box 3). Items related to conducting nutrition interventions had a low level of agreement across all rounds, particularly among dietitians and doctors. For example, only 18.8% of dietitians and 18.2% of doctors agreed that a medical professional should be able to ‘develop and appropriately document a nutrition care plan with specific goals’.

Box 3 **Consensus results on nutrition competencies for medicine**

*Items that achieved consensus (≥80% level of agreement)*

*Enabling competencies (knows)*

- Social determinants of health as they pertain to diet-related chronic disease.
- Basic scientific principles of human nutrition.
- Breast feeding and complementary feeding practices.
- How disease affects nutritional intake.
- How nutritional intake affects disease and recovery.
- Awareness of food allergies and intolerances, including when it is appropriate to refer for specialist intervention and support of advice provided as part of a multidisciplinary approach.
- The role and scope of practice of other health professionals in nutrition care (eg, dietitian, practice nurse).
- The role of other services in nutrition care, including awareness of the range of social and clinical prescribing options to support nutrition (eg, group education, emergency food provision or meal delivery services).

*Critical competencies (knows how)*

- Describe evidence-based dietary strategies for the promotion of health and prevention of disease.
- Identify when it is appropriate to refer to a specialist (eg, a dietitian).
- Locate and critique reputable sources of nutrition information.

*Application to practice (shows how/does)*

- Apply nutrition evidence appropriately in practice.
- Provide brief, evidence-based nutrition advice to patients.
- Refer at-risk patients or those who might benefit from specialist dietetics care.
- Work effectively in a multidisciplinary team to deliver nutrition care.
- Consider and apply principles of ethics related to nutrition care (eg, end of life feeding decisions).
- Demonstrate awareness of weight-based stigma.
- Demonstrate awareness of own personal health and nutrition biases.
- Demonstrate empathy and understanding in the context of nutrition care.
- Demonstrate awareness of the psychological, sociocultural determinants of health and how they might impact the dietary intake of individuals and populations.
- Select and apply dietary strategies in the promotion of health and prevention of disease.
- Demonstrate understanding of common medications and possible interactions with diet and nutrition.
- Consider the findings from nutrition screening and assessment as part of medical care.
- Coordinate care when an individual may benefit from further nutrition assessment or specialist dietary advice.
- Reinforce nutrition advice or recommendations provided by a specialist (eg, a dietitian).

#### Expert by experience panel

Forty-seven individuals based in Australia were invited to participate in the service user panel. Twenty-seven service users (57.5% response rate) completed the round 1 questionnaire, 19 completed round 2 and 16 completed round 3. Participants were mostly women and were aged between 21 and 69 years (table 5).

**Table 5:** Demographics of Delphi expert by experience panellists

Expert by experience panel			
Characteristics	Completed round 1 (n=27)	Completed round 2 (n=19)	Completed round 3 (n=16)
Age (years)			
Mean	32	33	35.25
Range	48 (21–69)	41 (24–65)	41 (24–65)
Gender			
Female	18 (69.2%)	11 (57.9%)	9 (56.3%)
Male	8 (30.8%)	7 (36.8%)	6 (37.5%)
Non-binary/prefer not to say	0	1 (5.3%)	1 (6.3%)
Level of education			
Primary education	1 (3.9%)	0	0
Secondary education	6 (23.1%)	6 (33.3%)	6 (37.50%)
Lower-degree tertiary education	9 (34.6%)	6 (33.3%)	6 (37.50%)
Higher-degree tertiary education	10 (38.5%)	6 (33.3%)	4 (25.00%)

Greater than 80% of the service user panel agreed that nutrition is relevant and important to medical care (Box 4). Items related to the provision of detailed nutrition had a low level of agreement across rounds and were eliminated (online supplemental materials). Almost all panellists (87.5%) agreed with the following statement in round 3: a medical professional (eg, a GP or other medical specialist) should be able to coordinate nutrition assessment, provide brief, individualised nutrition advice (which may include a nutrition education resource) and identify when to refer for specialist advice.

Box 4 **Consensus results: expert by experience panel**

*There was consensus among the expert by experience panel that*

- Nutrition is relevant to medical care.
- Nutrition is important in medical care.
- It is important that all doctors (GPs and other medical specialists) can identify a nutrition-related issue.
- It is important that all doctors (GPs and other medical specialists) can identify when to refer to a dietitian/nutritionist for specialist advice.
- It is important that other medical specialists (eg, a cardiologist) can locate and provide reputable nutrition education resources (eg, a pamphlet).

*Skills and attributes that achieved consensus:*

- Able to communicate effectively in the context of food and nutrition (eg, nutrition counselling, behaviour change strategies).
- Able to work in a team effectively to provide high-quality, effective nutrition care to patients.
- Demonstrates awareness of weight-based stigma and relationship with food and body.
- Is open-minded and willing to investigate nutrition-related concerns with a patient.
- Demonstrates confidence in ability to help a patient make changes to their diet.
- Demonstrates empathy and understanding in the context of food and nutrition.
- Demonstrates awareness of the sociocultural determinants of health (economic and social conditions that can underpin individual and group differences in health status) and how they might impact dietary intake of individuals and populations.

All nutrition-related skills and attributes achieved consensus in round 1 of the experts by experience panel (box 4). There was a particularly high level of agreement on items related to the ability to provide individualised advice and possess humanistic attributes.

## Discussion

Prior to this Delphi study, there has been no consensus based on this methodology on nutrition competencies for medical practice in Australia, New Zealand, the UK and Northern Ireland. A total of 25 nutrition competencies were validated by expert consensus, which may provide guidance to educators and regulators of medical education. The inclusion of a service user perspective validated the application of this competency framework to patient and family needs.

Competencies that achieved consensus through this Delphi process broadly fell into themes of team-based care, communication, professionalism and health promotion and disease prevention, in line with existing nutrition competencies for medicine internationally.^10^ While these themes include relevant nutrition context, they are broad areas of competency required across all aspects of medical care.^18 19^ This aligns with our previous work and other research suggesting the vertical integration of nutrition competencies into curricula based on cross-cutting themes, consistent with best practice for improving nutrition education.^10 20^ Existing curricula could be enhanced simply by applying broad medical competencies in a nutrition context, and successful examples of this have been implemented.^21^ Vertical integration of competencies limits the requirement for additional content to be incorporated into busy curricula, a frequently cited barrier to adequate nutrition education^22^ and has been shown to improve medical students’ clinical nutrition practice skills and perceptions of nutrition as a core element of total patient care.^23^ Pedagogies now emphasise active learning models, such as culinary exercises and simulation-based training with deliberate practice to promote knowledge translation and application.^24^

A lack of nutrition knowledge and skills has been found to extend to medical graduates and practising doctors and this deficit remains a barrier to nutrition care in practice,^25^ suggesting nutrition education should be integrated throughout the medical training process. Junior doctors have previously reported variable exposure to nutrition during practice experiences, and, in particular, variable experiences in the importance placed on nutrition by preceptors.^26^ For example, in a US study, medical students, residents and practising doctors reported witnessing very little nutrition counselling during shadowing experiences, and the nutrition information that was imparted was frequently outdated or incorrect.^27^ It is possible that an absence of experienced medical staff who can advocate for and model nutrition care limits this transfer. Future strategies to build the nutrition capacity of the medical workforce should consider upskilling medical educators in nutrition.

To coordinate nutrition care, GPs need the knowledge and skills to identify individuals who might benefit from a referral for dietetic advice and reinforce recommendations. Yet, core competency frameworks, including the RACGP curriculum and syllabus in Australia,^18^ and the UK Royal College of General Practitioners GP curriculum (2019) do not include any competencies relating specifically to nutrition.^28^ This may suggest the need to mandate nutrition education for GPs, specifically in their postentry-level programmes. Without incentive, it is unlikely that medical education providers will pursue curriculum changes that are not required. Accreditation standards provide an incentivised blueprint for curricula and therefore the inclusion of nutrition in such frameworks provides incentive for the integration of nutrition into medical curricula.^11^ The competencies identified in this study provide a benchmark for the knowledge and skills to be mandated.

Team-based approaches to nutrition care appear to be more successful than individual approaches, particularly when considering barriers doctors face, most notably, lack of time.^13 29^ Items related to conducting nutrition assessment and screening had a consistently low level of agreement across rounds. Practice nurses have been previously identified as the most appropriate health professional to conduct nutrition screening and this has been reported to enhance the quality of nutrition care provided.^30^ Further investigation into the role of non-medical professionals (eg, nurses) in the delivery of nutrition care, and the training required to support this, is warranted.

The results of the present study indicate that nutrition intervention, including counselling, may not be considered the responsibility of doctors. Interestingly, this is consistent with the results of a recent Delphi process, which defined objectives of undergraduate medical curricula in Latin America.^31^ This finding contradicts other nutrition competency frameworks, which recommend doctors provide nutrition intervention as part of care.^10^ The mean length of a GP consultation in Australia is only 13.9 min, and it is the norm for Australian GPs to manage multiple (3–4) patient-initiated problems per consultation.^32^ The Australian fee-for-service system may also discourage nutrition counselling by GPs, as it primarily rewards episodic care.^33^ Dietitians are highly qualified practitioners with specific expertise in counselling for behaviour change and health promotion.^34^ Yet, the rate of GP referrals to dietitians in Australia remains low.^32^ Having a dietitian on-site as part of a multidisciplinary clinic has been shown to increase the frequency of referral and facilitates message reinforcement in follow-up visits. Thus, there have been calls to increase the number of dietitians in primary care. Furthermore, GPs who have received nutrition training refer their patients more often to a dietitian.^35^ To this end, interprofessional nutrition education for medical and healthcare professionals may encourage more collaborative care in practice.

Person-centred care increases the likelihood that patients will adhere to lifestyle recommendations^36^ and conversely, a lack of person-centred care is associated with increased risk for conditions such as heart disease, high cholesterol and diabetes due to weight stigmatisation.^37^ Australian GPs have previously reported that they do not have sufficient knowledge and skills in nutrition to provide culturally, socially and economically sensitive nutrition care,^38^ and patients have previously reported a sense of dehumanisation in the healthcare system.^39^ Similar to the current work, the CanMEDS role of ‘Health Advocate’ encompasses competence to identify and address social determinants of health to achieve equity.^19^ To achieve this, nutrition education should be embedded within sociocultural frameworks to facilitate the delivery of person-centred nutrition care in practice. The inclusion of participatory nutrition education in medical training may promote awareness of social determinants of health and promote person-centred care. Community service opportunities, such as participation in a longitudinal nutrition education volunteer programme in underserved communities, have been associated with improved communication, interpersonal and leadership skills and higher empathy levels.^40^

### Strengths and limitations

While the Delphi method generates meaningful primary data from experts, there are limitations to this study. Despite efforts to recruit a diverse panel, participants were predominantly from Australia, and there was no participation from nurses, or dietitians from New Zealand, the UK or Northern Ireland. Furthermore, access to nutrition services and the level of involvement in nutrition care by different healthcare providers may vary between and within countries. As access to specialist nutrition services may impact the nutrition competencies required in practice, this may limit generalisability of the competencies defined in this study. We did not collect data on the work roles and settings of experts in healthcare practice, in particular, dietitians (eg, primary, or secondary care setting) and doctors (eg, access to specialised nutrition services) or assess differences in attributes of panellists who completed all rounds compared with those who did not. A larger response rate may have allowed greater confidence in the interpretation of findings, particularly as there was considerable diversity in the medical specialties included, and there was a high attrition rate in the expert by experience panel. Furthermore, while the modified Delphi process allowed for open-ended comments, and there were multiple rounds, the content of the round 1 survey was predetermined based on previous work and, therefore, may not capture the full extent of opinions. The lack of opportunity for discussion by the panellists between rounds could also be considered a limitation. Strengths of this study are its novelty, and that it attempted to engage a range of perspectives, including a service user perspective, and this diversity adds to the credibility of the framework for end users. The findings of this study can inform future work to further define the role and scope of practice of healthcare professionals in nutrition care, including the application of these competencies across different contexts. It is important to note that this is an initial consensus process and should be re-visited periodically.

## Conclusion

By consensus, this study defined 25 nutrition competencies for medicine. The service user panel identified an additional seven skills and attributes considered important in the receipt of nutrition care. This informs broad concepts and skills that may be taught in a nutrition context but could be included in other domains. The findings from this study suggest doctors need the knowledge and skills to consider the findings from nutrition screening and assessment, coordinate nutrition care when an individual may benefit from further assessment or intervention and provide support for advice provided by experts in nutrition as part of a multidisciplinary approach.

## Data Availability

All data relevant to the study are included in the article or uploaded as supplementary information.
