# Supplementary material for: Establishing consensus on nutrition competencies for medicine: a Delphi study
Source: BMJ Nutr Prev Health. 2024 Feb 5;7(1):68–77. doi: 10.1136/bmjnph-2023-000807 (PMC11221290; doi:10.1136/bmjnph-2023-000807)
Supplement: Supplementary data [file bmjnph-2023-000807supp001.pdf]

Supplementary Table 1. Expert panel: Level of agreement across rounds on nutrition competencies for medicine

**Statement**	**Level of agreement**			**Decision**
**Enabling Competencies (knows)**	**Round one**	**Round two**	**Round three**	
Social determinants of health as they pertain to diet-related chronic disease	89.6%	-	-	Included [1]
Nutritional content of food, including the major dietary sources of macronutrients and micronutrients	54.2%	71.8%	-	Eliminated [2]
Basic scientific principles of human nutrition	81.3%	-	-	Included
Nutrition requirements across the lifespan	75.0%	76.9%	-	Eliminated
Breastfeeding and complementary feeding practices	85.4%	-	-	Included
Awareness of food allergies and intolerances, including when it is appropriate to refer for specialist intervention and support of advice provided as part of a multidisciplinary approach	60.4%	71.8%	93.8%	Included
How disease affects nutritional intake	95.8%	-	-	Included
How nutritional intake affects disease and recovery	93.8%	-	-	Included
Nutrition screening (e.g., MST, MUST)	75.0%	64.1%	-	Eliminated
Nutrition Assessment	60.4%	46.2%	-	Excluded [3]
Awareness of behaviour change and counselling strategies to support dietary change	68.8%	69.2%	-	Eliminated
The role and scope of practice of other health professionals in nutrition care (e.g., dietitian, practice nurse)	97.9%	-	-	Included
The role of other services in nutrition care, including awareness of the range of social and clinical prescribing options to support nutrition (e.g., group education, emergency food provision or meal delivery services).	97.9%	-	-	Included
Demonstrate understanding of common medications and possible interactions with diet and nutrition	Suggested	87.2%	-	Included
Demonstrate understanding of the bi-directional relationship between food and health systems and environmental sustainability	Suggested	43.6%	-	Excluded
Critical Competencies (knows how)	Round one	Round two	Round three	Decision
Describe evidence-based dietary strategies for the promotion of health and prevention of disease	81.3%	-	-	Included
Describe evidence-based dietary strategies for the management of disease	79.2%	76.9%	-	Eliminated

Describe when to start nutrition support, including parenteral and enteral nutrition	66.7%	53.9%	-	Eliminated
Calculate energy expenditure of an individual	20.8%	12.8%	-	Excluded
Calculate nutrition requirements for an individual	20.8%	10.3%	-	Excluded
Take a diet/nutrition-related history	45.8%	53.9%	-	Eliminated
Use a validated tool to conduct nutrition screening (e.g., MST, MUST) and assessment (e.g., MNA, SGA)	66.7%	51.3%	-	Eliminated
Interpret findings from nutrition screening and assessment	70.8%	56.4%	-	Eliminated
Identify and define nutritional problems	66.7%	71.8%	-	Eliminated
Identify when it is appropriate to refer to a specialist (e.g., a dietitian)	100.0%	-	-	Included
Locate reputable nutrition information	91.7%	-	-	Included
Application-based competencies (shows how/does)	Round one	Round two	Round three	Decision
Conduct nutrition screening as part of routine medical care	77.1%	69.2%	-	Eliminated
Conduct nutrition assessment as part of routine medical care	52.1%	35.9%	-	Excluded
Apply nutrition evidence appropriately in practice	85.4%	-	-	Included
Select and prescribe dietary strategies in the prevention and management of disease	62.5%	Separated	-	-
Select and apply dietary strategies in the promotion of health and prevention of disease	-	81.6%	-	Included
Select and apply dietary strategies in the treatment of disease	-	68.4%	-	Eliminated
Provide brief, evidence-based nutrition advice to patients	85.4%	-	-	Included
Provide evidence-based nutrition education to patients	56.3%	53.9%	-	Eliminated
Develop and appropriately document a nutrition care plan with specific goals	25.0%	23.1%	-	Excluded
Refer at-risk patients or those who might benefit from specialist dietetic care	97.9%	-	-	Included
Provide nutrition counselling using a range of behaviour change techniques (e.g., motivational interviewing) to elicit positive nutrition behaviour change	54.2%	51.3%	-	Eliminated
Monitor nutrition status	64.6%	59.0%	-	Eliminated
Modify dietary recommendations or a nutrition care plan as needed	31.3%	35.9%	-	Excluded
Work effectively in a multidisciplinary team to deliver nutrition care	87.5%	-	-	Included
Initiate nutrition support when appropriate	58.3%	59.0%	-	Eliminated
Consider and apply principles of ethics related to nutrition care (e.g., end of life feeding decisions)	87.5%	-	-	Included

Demonstrate awareness of weight-based stigma	89.6%	-	-	Included
Demonstrate awareness of own personal health and nutrition biases	87.5%	-	-	Included
Demonstrate empathy and understanding in the context of nutrition care	93.8%	-	-	Included
Demonstrate awareness of the socio-cultural determinants of health and how they might impact dietary intake of individuals and populations	93.8%	-	-	Included
Demonstrate confidence in ability to elicit nutrition behaviour change in patients	58.3%	41.0%	-	Excluded
Identifies opportunities and advocates for change to the wider social, cultural and/or political environment to improve nutrition	Suggested	48.7%	-	Excluded
Identify the impact of mental health on diet and disease and refer to mental health specialists as appropriate	Suggested	76.9%	-	Eliminated
Take a brief diet history as part of a medical examination	-	-	72.3%	Eliminated
Allocate nutrition screening to another member of the multidisciplinary team (e.g., a GP practice nurse)	-	-	68.1%	Eliminated
Consider the findings from nutrition screening and assessment as part of medical care	-	-	87.2%	Included
Coordinate care when an individual may benefit from further nutrition assessment or specialist dietary advice	-	-	87.2%	Included
Reinforce nutrition advice or recommendations provided by a specialist (e.g., a dietitian)	-	-	89.36%	Included

[1] Included = Included by consensus (defined as ≥80% level of agreement)
[2] Eliminated = Did not achieve consensus
[3] Excluded = Excluded by consensus (defined as ≥50% level of disagreement or ≤50% level of agreement)

Supplementary Table 2. Importance and relevance of nutrition to medical care

Statement	Level of agreement (%)		
	Round one	Round two	Round three
How important is nutrition to your medical care?	77.8%	89.5%	-
How relevant is nutrition to your medical care?	81.5%	-	-

Supplementary Table 3. Relevance of skills and attributes in nutrition care

Nutrition skills and attributes for medicine	Level of agreement
Able to communicate effectively in the context of food and nutrition (e.g., nutrition counselling, behaviour change strategies)	92.00%
Able to work in a team effectively to provide high-quality, effective nutrition care to patients	84.00%
Demonstrates awareness of weight-based stigma and relationship with food and body	96.00%
Is open-minded and willing to investigate nutrition-related concerns with a patient	96.00%
Demonstrates confidence in ability to help a patient make changes to their diet	88.00%
Demonstrates empathy and understanding in the context of food and nutrition	100.00%
Demonstrates awareness of the socio-cultural determinants of health (economic and social conditions that can underpin individual and group differences in health status) and how they might impact dietary intake of individuals and populations	92.00%

BMJ Publishing Group Limited (BMJ) disclaims all liability and responsibility arising from any reliance placed on this supplemental material which has been supplied by the author(s)

Supplementary Table 4. The role of general practitioners and other medical specialists in nutrition care

Statement	Level of agreement (%)	
	**Round one**	**Round two**
How important is it to you that a GP can assess your diet?	73.1%	79.0%
How important is it to you that a GP can identify nutrition-related issues?	84.6%	-
How important is it to you that a GP can provide brief nutrition advice?	69.2%	79.0%
How important is it to you that a GP can provide detailed nutrition advice?	65.4%	57.9%
How important is it to you that a GP can provide individualised nutrition advice?	69.2%	57.9%
How important is it to you that a GP can locate and provide you with nutrition education resources (e.g., a pamphlet)?	73.1%	79.0%
How important is it to you that a GP can manage a nutrition-related health condition?	73.1%	79.0%
How important is it to you that a GP can identify when to refer to a dietitian/nutritionist for specialist advice?	88.5%	-
How important is it to you that a medical specialist can assess your diet?	76.0%	73.7%
How important is it to you that a medical specialist can identify nutrition-related issues?	76.0%	84.2%
How important is it to you that a medical specialist can provide brief nutrition advice?	60.0%	73.7%
How important is it to you that a medical specialist can provide detailed nutrition advice?	64.0%	63.2%
How important is it to you that a medical specialist can provide individualised nutrition advice?	64.0%	73.7%
How important is it to you that a medical specialist can locate and provide you with reputable nutrition education resources (e.g., a pamphlet)?	68.0%	84.2%
How important is it to you that a medical specialist can manage a nutrition-related health condition?	68.0%	63.2%
How important is it to you that a medical specialist can identify when to refer to a dietitian/nutritionist for specialist advice?	80.0%	-
